# Coral Colonisation of an Artificial Reef in a Turbid Nearshore Environment, Dampier Harbour, Western Australia

**DOI:** 10.1371/journal.pone.0075281

**Published:** 2013-09-10

**Authors:** David Blakeway, Michael Byers, James Stoddart, Jason Rossendell

**Affiliations:** 1 Marine Research, MScience Pty Ltd, Nedlands, western Australia, Australia; 2 Coastal Operations, Rio Tinto iron Ore, Dampier, western Australia, Australia; University of New South Wales, Australia

## Abstract

A 0.6 hectare artificial reef of local rock and recycled concrete sleepers was constructed in December 2006 at Parker Point in the industrial port of Dampier, western Australia, with the aim of providing an environmental offset for a nearshore coral community lost to land reclamation. Corals successfully colonised the artificial reef, despite the relatively harsh environmental conditions at the site (annual water temperature range 18-32°C, intermittent high turbidity, frequent cyclones, frequent nearby ship movements). Coral settlement to the artificial reef was examined by terracotta tile deployments, and later stages of coral community development were examined by in-situ visual surveys within fixed 25 x 25 cm quadrats on the rock and concrete substrates. Mean coral density on the tiles varied from 113 ± 17 SE to 909 ± 85 SE per m^2^ over five deployments, whereas mean coral density in the quadrats was only 6.0 ± 1.0 SE per m^2^ at eight months post construction, increasing to 24.0 ± 2.1 SE per m^2^ at 62 months post construction. Coral taxa colonising the artificial reef were a subset of those on the surrounding natural reef, but occurred in different proportions— *Pseudosiderastreatayami* *,* *Mycediumelephantotus* and *Leptastrea* *purpurea* being disproportionately abundant on the artificial reef. Coral cover increased rapidly in the later stages of the study, reaching 2.3 ± 0.7 SE % at 62 months post construction. This study indicates that simple materials of opportunity can provide a suitable substrate for coral recruitment in Dampier Harbour, and that natural colonisation at the study site remains sufficient to initiate a coral community on artificial substrate despite ongoing natural and anthropogenic perturbations.

## Introduction

**Figure 1:**
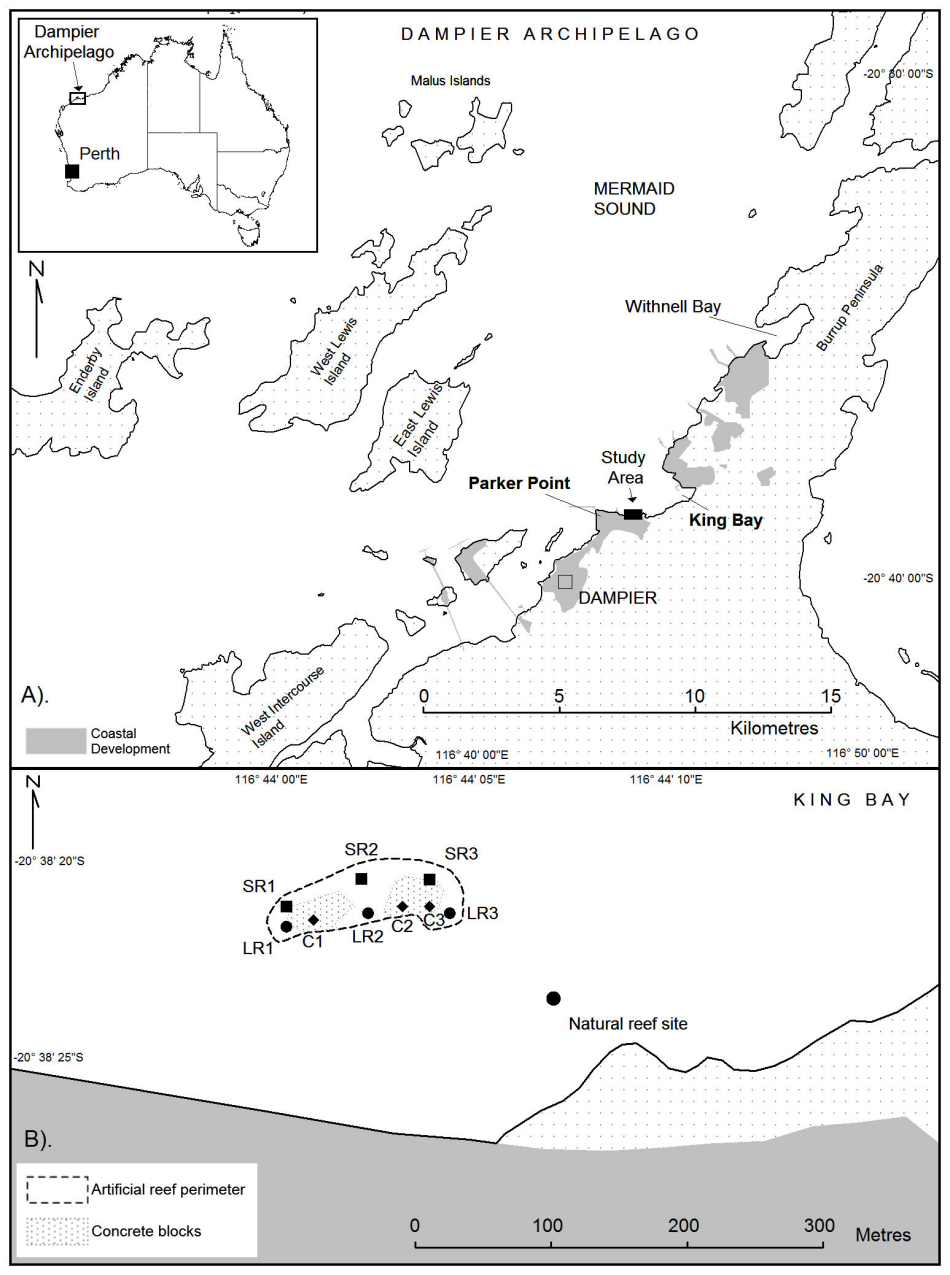
General location map of Dampier Harbour, Western Australia (A). Areas of coastal development are shaded in grey, including wharves, jetties, causeways, processing plants and stockpiles. Enlargement of the study area (B), showing the location and configuration of the artificial reef, the nine subsites on the reef, and a nearby natural reef site where water quality and coral community composition data were collected. Subsites on the artificial reef are named according to their aspect (S = seaward, L = landward), substrate (R = rock, C = concrete) and location (1, 2 and 3 = west, central, east). Quadrat surveys were undertaken at all nine subsites. Tile deployments were undertaken at the six rock substrate subsites, and six subsites at the natural reef site.

The *Pilbara* coast of northwestern Australia is located between 22° S 114° E and 20° S 119° E, in a semi-arid environment with a range of coastal habitats including rocky shores, sandy shores, mud flats and mangroves [1]. The Port of Dampier is situated midway along the *Pilbara* coast in the inner Mermaid Sound, a rock-dominated area with a complex coastline and numerous islands (Figure 1). Dampier’s marine environment is naturally dynamic, with a tidal range of up to 5 m [2], an annual water temperature range of approximately 18-32°C [2,3], high ultraviolet radiation [4] and high turbidity derived from both natural and anthropogenic causes [5]. Cyclones are frequent and often bring destructive waves, freshwater runoff, extreme turbidity and sedimentation [3,5–8].

Following its original development for iron ore export in the mid 1960s, the port now handles bulk shipments of iron ore, liquefied natural gas, salt and general cargo, as well as providing a supply base for offshore oil and gas operations [9]. Consequently, marine habitats in Dampier Harbour are subject to anthropogenic disturbance from ship traffic, dredging programs and land reclamation [10,11]. Modification of nearshore habitat for infrastructure development has removed sections of the original coral communities and possibly altered the composition and cover of nearby communities [12].

Coral communities occur on most subtidal hard substrates in Dampier Harbour [13]. Species diversity in these communities is moderately high and live coral cover is commonly between 10 and 40% [14]. However there is little true biogenic reef development in the harbour [1], suggesting that the natural positive and negative contributions to reef accretion are closely balanced. If this is the case, existing coral communities may be vulnerable to any additional negative natural or anthropogenic influences. In this context, coral settlement and recruitment are likely to be key processes determining the persistence of Dampier’s coral communities and also their capacity to recover from disturbance.

In December 2006, an artificial reef was constructed within the inner Dampier Harbour as an environmental offset for a nearshore coral community lost to land reclamation for the expansion of an ore stockyard. While it would have been preferable to expand the stockyard landward, that was constrained by steep rocky ground and an existing rail line. Given the history of coral loss in the inner harbour, creation of new coral habitat in an area of soft substrate was considered by environmental managers and regulators to be an appropriate offset contribution, in conjunction with the management-related knowledge acquired during the project.

The artificial reef was constructed from two locally-sourced recycled materials: rock boulders from a dismantled seawall and concrete foundation sleepers from a disused conveyor. The boulders were approximately 1-2 m diameter and the concrete sleepers were 2.5 x 0.7 x 0.5 m. The reef was located approximately 200 m from shore on a flat sand substrate at a depth of approximately 6.5 m at mean sea level (MSL), 4 m on lowest astronomical tide (LAT) and 9 m on highest astronomical tide (HAT).

The artificial reef was surveyed at six month intervals between August 2007 and February 2012 to determine the level of coral settlement to tiles on the artificial reef and nearby natural reef, and the recruitment and growth of corals on the artificial reef. The intent of the monitoring was to test whether a coral community would develop on the reef to a targeted minimum cover of 10% within 10 years, and to describe coral recruitment and growth processes to help predict coral recovery rates within the broader area of the inner Dampier Harbour.

## Materials and Methods

### Ethics statement

The work described in this article was undertaken in accordance with Permit SD2005/0028 issued on the 22nd of March 2006 by the Australian Government Department of the Environment and Heritage.

### Water quality

Water temperature and turbidity were logged at a natural reef site (23°38.37’ S, 116°44.31’ E) 100 m southeast of the artificial reef during various environmental monitoring programs unrelated to the artificial reef project. The loggers were placed among coral colonies at a depth of 4.5 m MSL. Water temperature was recorded hourly between February 2008 and February 2010 with a TidBiT V2 water temperature data logger, and turbidity was recorded (in nephelometric turbidity units - NTU) at 30 minute intervals between November 2007 and August 2010 with either an SAS [15] or Wetlabs ECO-NTU logger. The relationship between NTU and total suspended solids (TSS) was not investigated at this site, but work elsewhere in Dampier Harbour has shown TSS to be commonly between 1.0 and 1.5 mg/L per NTU [16].

### Settlement tiles

Coral settlement was sampled by deploying *terra* *cotta* tiles (11 x 11 x 1 cm) at six subsites across the artificial reef (Figure 1) and six subsites at the natural reef site. Tile deployments were undertaken in four consecutive years, 2008 to 2011. In the first four deployments, tiles were deployed in February or March, two to four weeks before the predicted primary coral spawning period in each year [17], and retrieved approximately three months after the spawning period. The fifth deployment was undertaken in September 2011, with retrieval five months later in February 2012, to examine settlement after the secondary spawning period in spring [17,18].

In each deployment, twelve tiles per subsite were attached to concrete Besser blocks (three blocks x 4 tiles per block) with stainless steel screws and 10 mm PVC spacers. Besser blocks were used as the attachment medium because the rock substrate of the artificial reef was too hard to drill for direct tile attachment. The three blocks at each subsite were closely spaced and oriented horizontally.

On retrieval, tiles were bleached in a 10% NaOH solution for 24 hours, rinsed in fresh water, dried and examined under a dissecting microscope [19]. Corals were classified into four categories: Acroporidae, Pocilloporidae, Poritidae and ‘other’, based on reference images in Babcock et al. [19]. Recruits from all tiles on a block were pooled as a single replicate and the resultant total transformed as *ln*(*x+1*). A two-way analysis of variance was undertaken on the artificial reef data, testing the effect of survey and aspect (seaward v. landward) on coral density.

### Quadrats

Coral recruitment was sampled by in-situ visual inspection within fixed 25 x 25 cm quadrats. Before the first survey in August 2007, small concrete lugs were cemented to the substrate as a means of positioning the quadrats consistently in each survey. 162 quadrats were distributed equally amongst the nine subsites shown in Figure 1. Subsites were named according to their aspect (S = seaward or L = landward), substrate type (R = rock or C = concrete) and location (numbers 1, 2 and 3 corresponding respectively to west, central and east locations on the reef). Each subsite comprised three boulders or sleepers, each with six quadrats; three on the horizontal surface and three on the vertical surface. In each survey, the quadrats were photographed and inspected visually at close range for juvenile corals. Corals were counted and classified taxonomically, to the extent possible. Regular cross checks between observers were undertaken to ensure taxonomic classifications matched and counts remained within ± 20% at each site. The maximum diameter of each colony was measured using plastic calipers. Percent coral cover was derived by summing the area of coral per quadrat (calculated assuming circular colony shape) and dividing by the quadrat area. Using the maximum colony diameters to calculate area will have overestimated percent cover, but as most colonies were approximately circular the difference will be slight and we have not corrected for it.

Two three-way analyses of variance were undertaken on the quadrat data. The first examined the effects of substrate type, substrate orientation and location (i.e. west, central, east) on recruit density on the landward side of the reef and the second examined the effect of aspect, substrate orientation and location on recruit density on the rock substrate. It was not possible to combine all four factors in a single analysis because the concrete substrate was restricted to the landward side of the reef (Figure 1B). Analyses were undertaken on pooled counts of coral recruits on each of the three rock boulders or concrete sleepers at each subsite. The pooled raw data were normalised with a *ln*(*x+1*) transformation.

### Natural reef

Patches of coral reef occur to the north, east, and southeast of the artificial reef. Coral cover and taxonomic composition were surveyed in the southeast coral patch, adjacent to the water quality instruments, during environmental monitoring programs unrelated to the artificial reef project. The site is 100m from the artificial reef (Figure 1) at a depth of 4.5 m MSL. The substrate at this site is typical of fringing reefs in the harbour, consisting of bedrock (Gidley granophyre) and bedrock boulders, interspersed with siliciclastic and carbonate sand and gravel.

Estimates of coral cover and composition at the natural reef site were derived from point count analysis of digital still images along five fixed 10 m x 1 m transects, at 30 images per transect and 30 points per image. Hard coral colonies were identified to genus and counted from the images. Care was taken to ensure corals spanning more than one image were only counted once.

## Results

### Water quality

**Figure 2:**
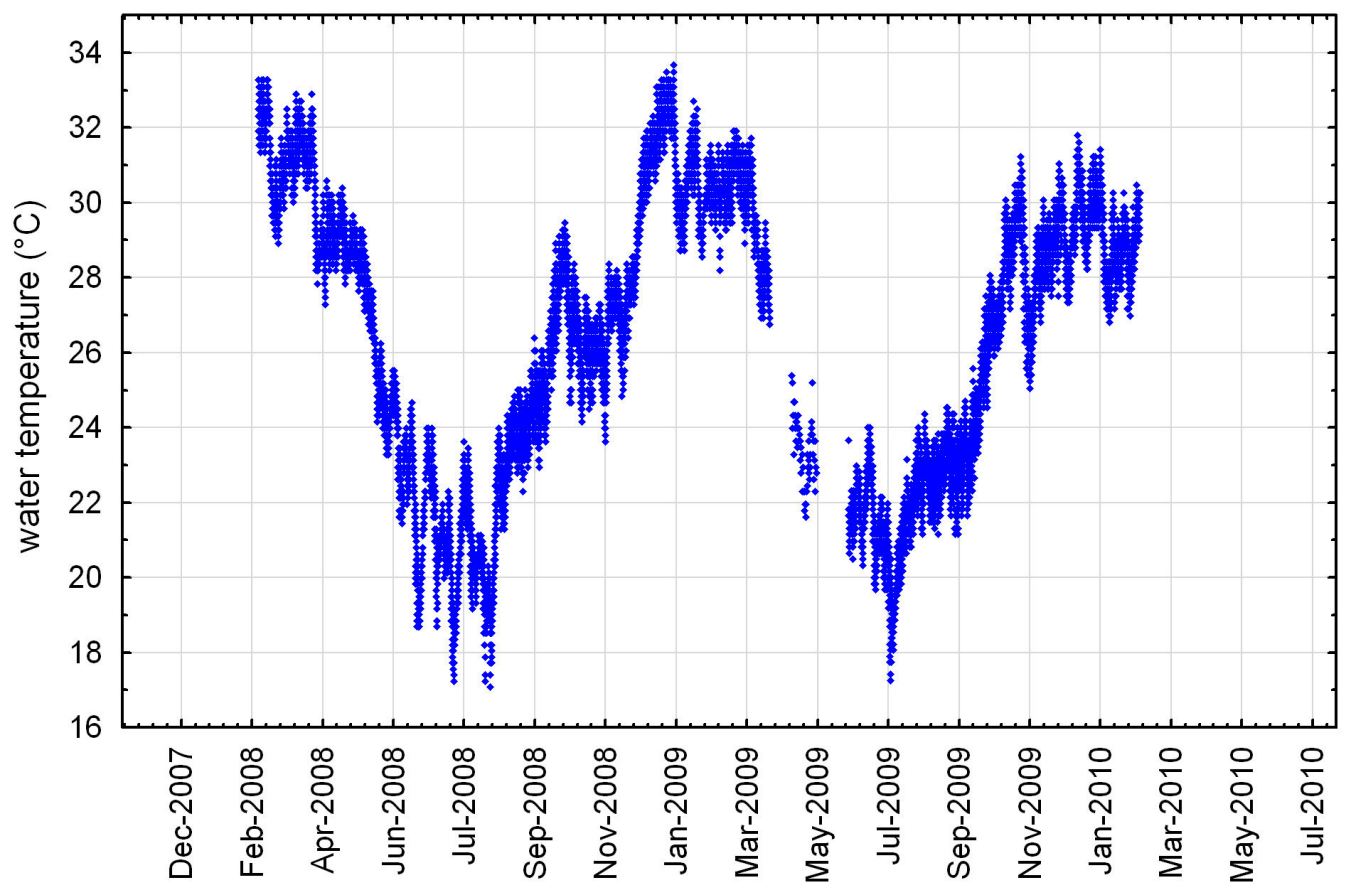
Water temperature at the natural reef site, February 2008 to February 2010 (see Figure 1 for location).

The natural reef site underwent marked seasonal temperature variation, with seasonal minima of approximately 19°C in July and maxima of approximately 32°C in February (Figure 2), consistent with the seasonal pattern described by Pearce et al. [2]. Low and high temperature extremes at the site over the two year period were 17.1 and 33.7°C respectively. In early 2008, water temperatures above 32°C for more than two weeks caused bleaching in more than half the colonies at the site, although virtually all recovered and regained colour within three months [16].

**Figure 3:**
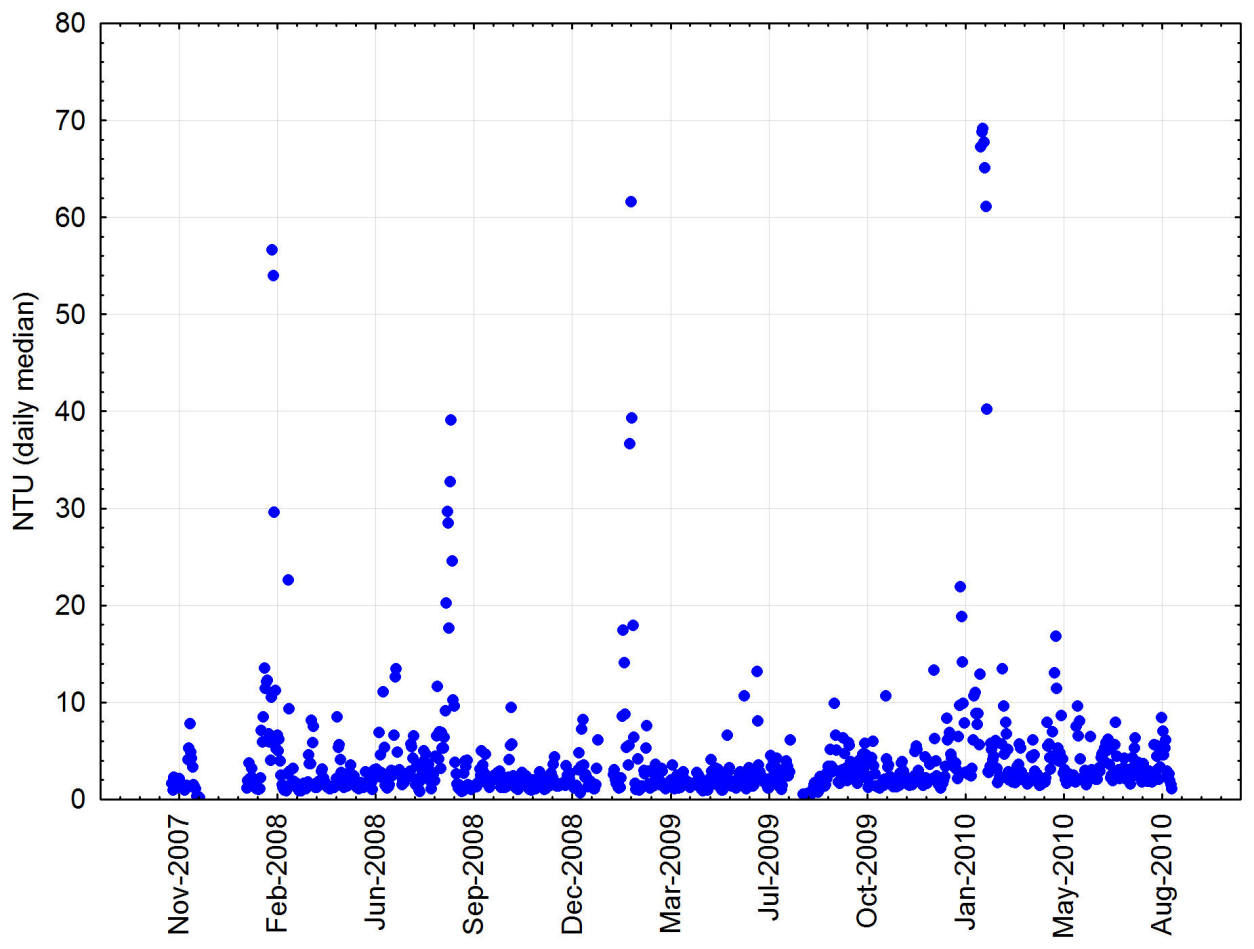
Median daily turbidity in nephelometric turbidity units (NTU) at the natural reef site, November 2007 to August 2010 (see Figure 1 for location). No data were recorded in December 2007 and January 2008.

Turbidity at the natural reef site was highly variable. Although the average daily median turbidity over the 31 months of records was only 2.4 NTU, many high turbidity events were recorded, with daily median turbidity often above 10 NTU and occasionally exceeding 50 NTU (Figure 3).

### Artificial Reef

The rock and concrete substrates of the artificial reef proved stable over time, remaining in position despite several cyclones affecting the area over the 2006-2012 study period. Turf algae and barnacles were conspicuous early colonisers, followed by encrusting sponges, colonial and solitary ascidians, macroalgae (primarily *Lobophora* *variegata* ) and coralline algae. A ubiquitous layer of fine sediment, bound by turf algae, covered the horizontal surfaces of the artificial reef to a depth of one to six mm. Despite this sediment cover, corals were present on the reef within the first few months after construction, although they remained inconspicuous until the second year. The artificial reef was also colonised by a diverse fish community, initially composed primarily of migrants from surrounding reefs but later including juveniles recruiting directly to the reef [20].

### Settlement tiles

#### Coral abundance and distribution

**Figure 4:**
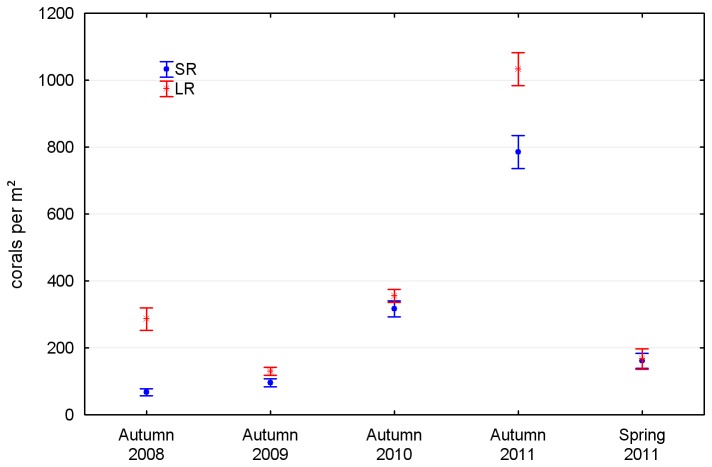
Variation in coral settlement density on tiles at the artificial reef 2008-2012: SR – seaward sites, rock substrate LR – landward sites, rock substrate.

Coral abundance on the artificial reef settlement tiles was relatively low in each of the 2008–2010 autumn surveys (mean density 113 to 336 corals per m^2^), with a pulse to 909 per m^2^ in autumn 2011 followed by a reduction to 164 per m^2^ in spring 2011. The density of settled corals showed high interannual variability, with greater settlement to tiles on the landward side of the reef than the seaward side, although the magnitude of the difference varied between years (Table 1, Figure 4).

**Table 1:** Results of the analysis of variance testing the significance of variations in coral density on tiles between years and aspects (seaward vs. landward).

**Effect**	**SS**	**DF**	**MS**	***F***	***p***
Year	43.64	4	10.91	54.76	<0.001
Aspect	2.49	1	2.49	12.48	0.001
Year*[Aspect]	3.17	4	0.79	3.97	.006

#### Taxonomy

**Figure 5:**
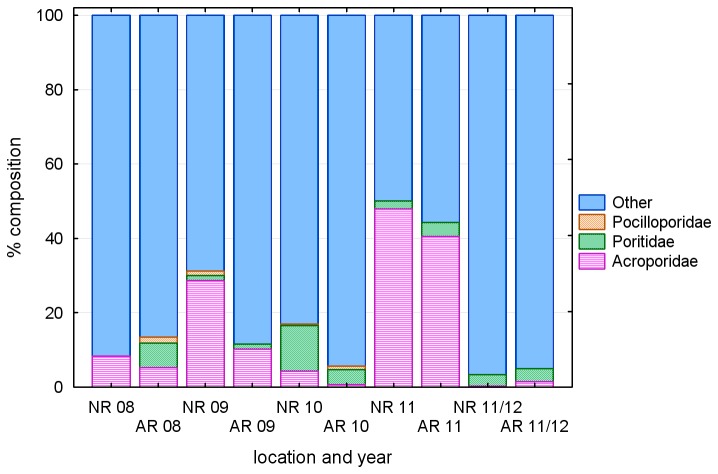
Percentage composition of corals settling on tiles at the artificial reef (AR) and natural reef (NR) in the five deployments undertaken from 2008 to 2011/12.

The most abundant category on the artificial reef in the autumn spawning surveys was “other” accounting for more than 85% of recruits in 2008-2010, and more than 55% in 2011 (Figure 5). Of the corals that were identifiable to family, Acroporidae and Poritidae recruits were recorded on the artificial reef in all autumn spawning surveys. Acroporidae were particularly abundant in the 2011 autumn, accounting for ~40% of the total. Pocilloporidae were absent in the 2009 and 2011 autumn surveys. The spring 2011 survey was dominated by corals in the ‘other’ category. Poritidae and Acroporidae were present in low numbers, and no Pocilloporidae were recorded. The taxonomic composition at the natural reef site was similar to that of the artificial reef in all surveys with the exception of the absence of Acroporidae and Pocilloporidae at the natural reef site in autumn 2008 (Figure 5).

### Quadrats

#### Coral abundance and distribution

**Figure 6:**
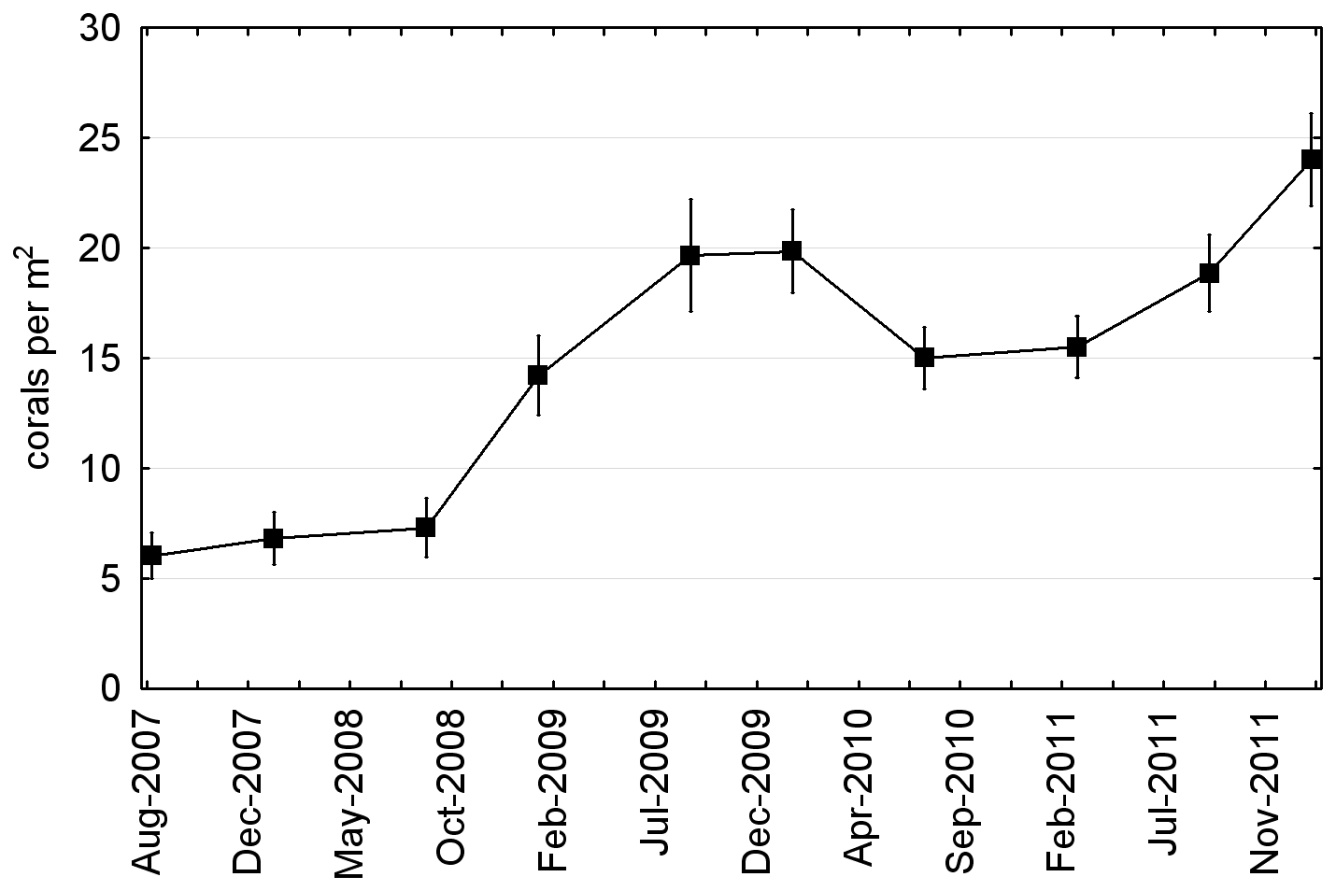
Mean ± SE coral density (all subsites combined) on the artificial reef between 2007 and 2012.

Coral abundance in the quadrats was initially low and increased slowly; mean density (± SE) in the 162 quadrats on the artificial reef was 6.0 ± 1.0 per m^2^ at eight months post construction, 6.8 ± 1.8 per m^2^ at 14 months post construction, and 7.3 ± 1.3 per m^2^ at 21 months post construction (Figure 6). In subsequent surveys coral abundance fluctuated considerably but maintained an overall positive trend, reaching 24.0 ± 2.1 per m^2^ in the final survey in February 2012. Coral distribution was spatially clumped at the quadrat scale (variance-to-mean ratio 1.86 in February 2012), due primarily to aggregations of the most abundant species *Pseudosiderastreatayami* .

**Figure 7:**
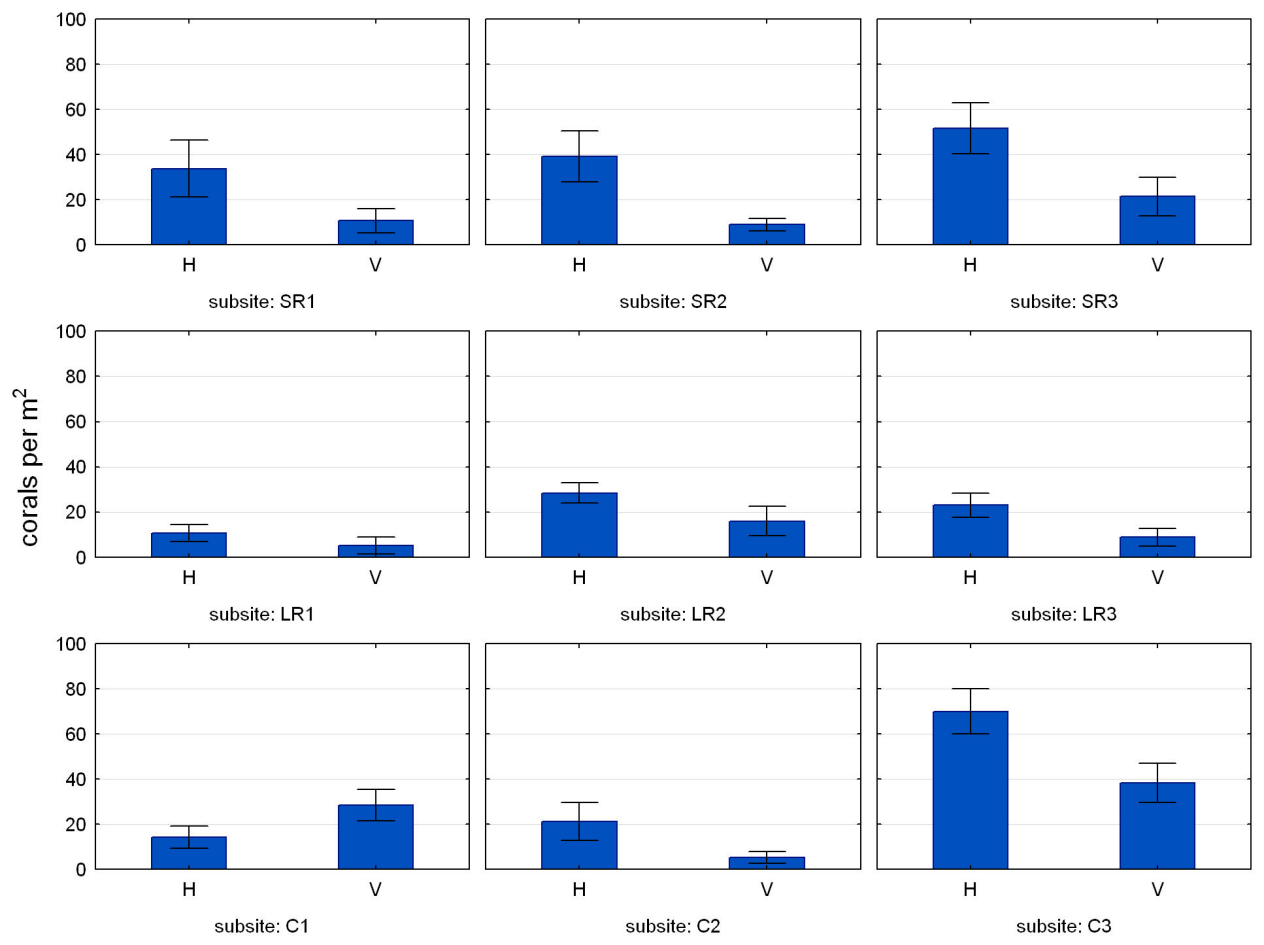
Mean ± SE coral density on horizontal (H) and vertical (V) surfaces across the nine subsites on the artificial reef in February 2012. See Figure 1B for subsite locations.

**Figure 8:**
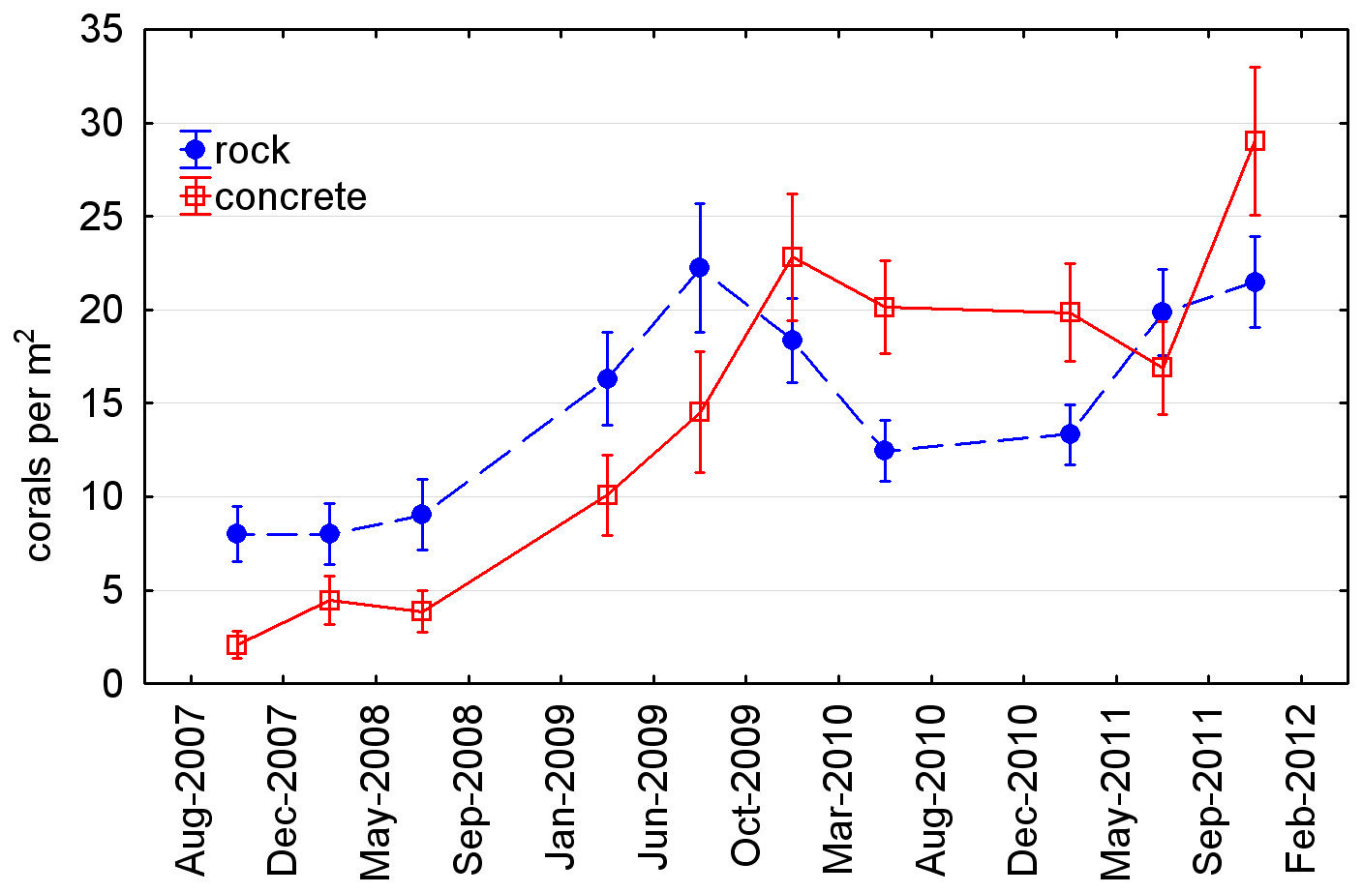
Mean ± SE coral density over time on the landward rock (LR) and concrete substrates of the artificial reef. Seaward rock substrates were not included in this figure because there is no corresponding concrete substrate on the seaward side of the reef.

Figure 7 shows mean coral density at each of the nine subsites across the reef in February 2012. Results of the statistical tests on coral density across these subsites are presented in Table 2. Significant differences in coral density occurred between substrates (concrete > rock), aspects (seaward > landward), and surface orientations (horizontal > vertical). The effect of location on coral density was significant on the landward side of the reef (east and central > west) but not the seaward side. There was a significant interaction between location and orientation on the landward side, due to the unusually high coral density on vertical surfaces at subsite C1 (Figure 7). All these differences were apparent from the outset of the study, with the exception of the greater density on concrete than rock, which only became apparent in the later surveys (Figure 8). Also apparent from the outset was a greater abundance of non-coral invertebrates—primarily encrusting sponges—on vertical surfaces than horizontal, although this difference was not quantified or tested.

**Table 2:** Results of analyses of variance testing the significance of variations in coral density in quadrats on the landward side of the artificial reef and on the rock substrate.

***QuadratsonLandwardSideofArtificialReef***					
**Effect**	**SS**	**DF**	**MS**	***F***	***p***
Substrate	76.4	1	76.44	19.34	<0.001
Location	122.4	2	61.19	15.49	<0.001
Orientation	43.5	1	43.54	11.02	0.003
Substrate*Location	136.5	2	68.23	17.27	<0.001
Substrate*Orientation	0.4	1	0.44	0.11	0.743
Location*Orientation	47.8	2	23.91	6.05	0.007
Substrate*Location*Orientation	23.4	2	11.69	2.96	0.071
***QuadratsonRockSubstrateOnly***					
**Effect**	**SS**	**DF**	**MS**	***F***	***p***
Aspect	46.70	1	46.70	5.22	0.031
Location	27.70	2	13.90	1.55	0.233
Orientation	117.40	1	117.40	13.12	0.001
Aspect*Location	19.10	2	9.50	1.07	0.360
Aspect*Orientation	23.40	1	23.40	2.61	0.119
Location*Orientation	4.10	2	2.00	0.23	0.799
Aspect*Location*Orientation	0.10	2	0.00	0.00	0.997

#### Taxonomy

*Pseudosiderastreatayami* was the most abundant coral species identified on the artificial reef, comprising 30% of the 243 colonies recorded in the final survey. Other common species included *Turbinariamesenterina* (14%), *Mycediumelephantotus* (6.6%) and *Leptastrea* *purpurea* (6.2%). Several faviid genera were present in proportions of less than 5% each, including *Cyphastrea*, *Goniastrea*, *Favia* and *Favites*. Juvenile (attached stage) fungiids constituted 4% of the total. Additional genera recorded in lower numbers (<5 but >2 individuals) included *Acropora*, *Acanthastrea*, *Psammocora*, and *Heteropsammia*. Several additional genera were recorded as only one or two individuals.

#### Colony size and percent cover

**Figure 9:**
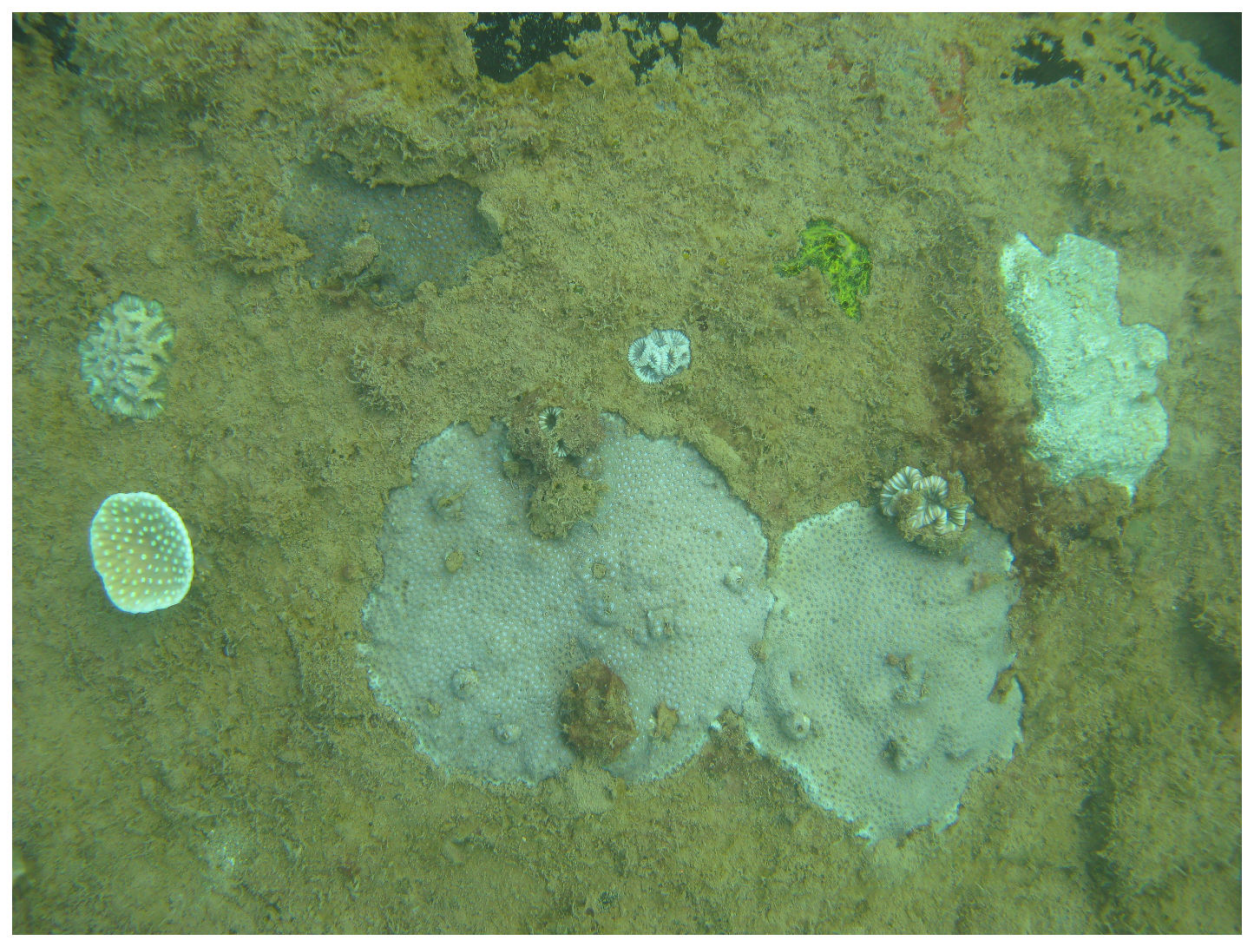
Spreading growth habit of two *Leptastrea* *purpurea* colonies on the artificial reef (centre of photo, larger colony approximately 130 mm diameter).

In the first survey at eight months post construction, the median colony diameter was one mm and the maximum diameter was six mm. Median colony diameter increased slowly, due to the presence of new recruits in each survey, reaching nine mm in the final survey at 62 months post construction. Mean colony diameter increased steadily, reaching 21 mm in the final survey. The fastest-growing coral species was the faviid *Leptastrea* *purpurea* , with radial extension rates averaging 10-15 mm per year. The largest colony recorded in the final survey was a *L* *. purpurea* colony of 152 mm diameter. A few larger colonies were found outside the quadrats, including *Leptastrea*, *Cyphastrea* and *Porites* colonies of more than 200 mm diameter. The growth pattern of these genera was typically flat and spreading; even the largest colonies appeared to be less than 10 mm thick (Figure 9).

**Figure 10:**
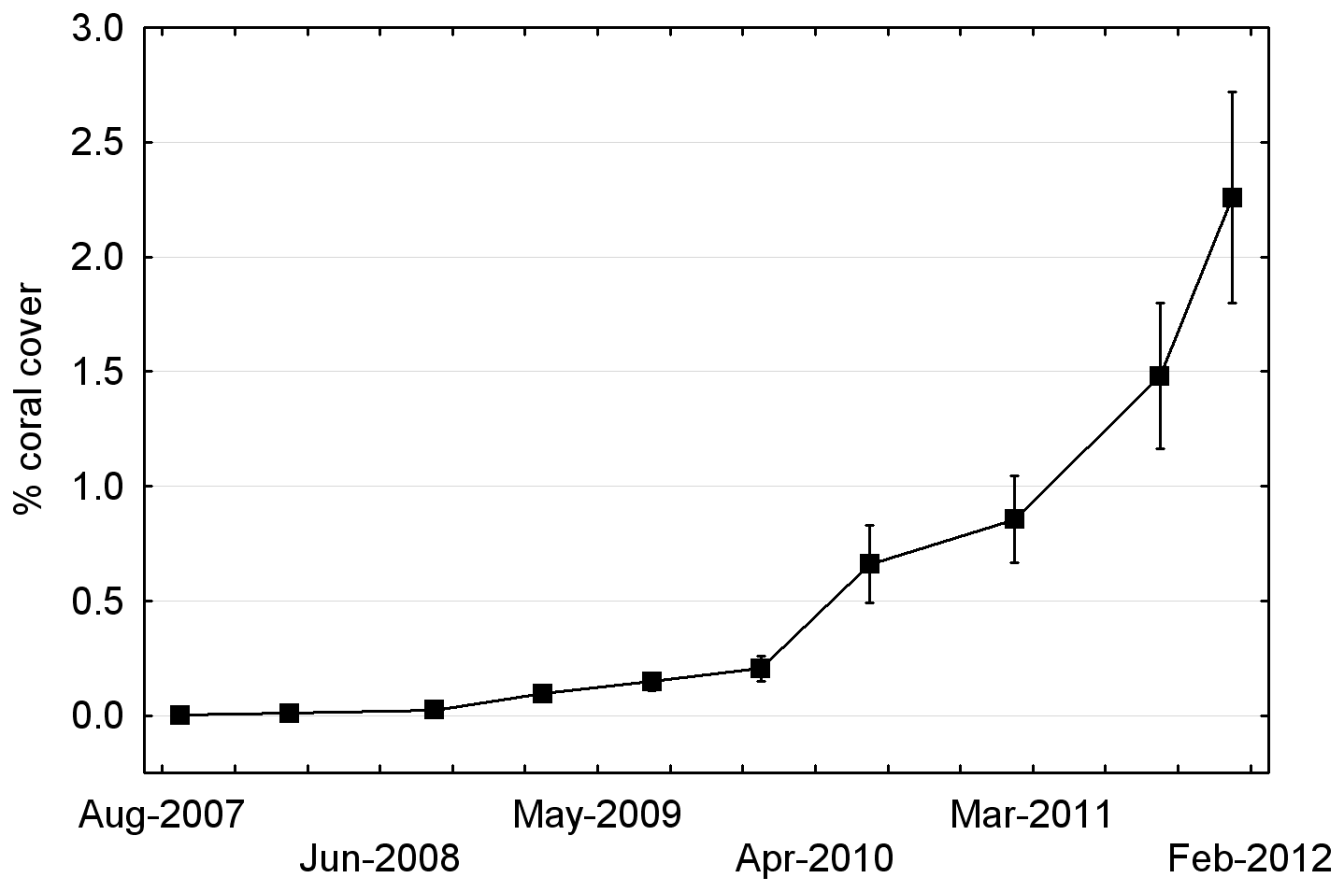
Mean ± SE coral cover (all subsites combined) on the Artificial Reef between 2007 and 2012.

Mean coral cover on the artificial reef rose slowly in the first couple of years but increased rapidly in the latter half of the study, reaching an estimated 2.3 ± 0.7 SE % in the final survey (Figure 10). *Leptastrea* *purpurea* was the greatest contributor to coral cover, due to its abundance and high extension rate. Mean coral cover was greater on vertical surfaces (2.7 ± 0.6 SE %) than horizontal surfaces (1.8 ± 0.8 SE %), despite the lower mean coral density on vertical surfaces.

### Natural reef

The coral community at the natural reef site occurs as a discontinuous veneer over the subtidal hard substrate, with mean live cover of approximately 30-35% between 2006 and 2010, as assessed from belt transects. Common coral families at the site, in order of numerical abundance, were Faviidae (57%), Dendrophyllidae, Poritidae and Mussidae. *Favites* was the most abundant coral genus, comprising 24.3% of the 531 colonies recorded in the final survey (Table 3). Other common genera included *Turbinaria* (16%), *Favia* (10%), *Platygyra* (9.6%) and *Goniastrea* (9%). In contrast to their abundance on the artificial reef, *Pseudosiderastrea, Leptastrea* and *Mycedium* accounted for only 1.9%, 1.7% and 0.6%, respectively, of recorded colonies on the natural reef transects.

**Table 3:** Coral genera and number of colonies along five ten metre transects at the natural reef site.

**Genus**	**Count**	**Percentage (%)**
*Favites*	129	24.3
*Turbinaria*	85	16.0
*Favia*	53	10.0
*Platygyra*	51	9.6
*Goniastrea*	48	9.0
*Porites*	38	7.2
*Lobophyllia*	23	4.3
*Pavona*	23	4.3
*Hydnophora*	11	2.1
*Merulina*	11	2.1
*Pseudosiderastrea*	10	1.9
*Leptastrea*	9	1.7
*Cyphastrea*	7	1.3
*Galaxea*	7	1.3
*Montipora*	7	1.3
*Symphyllia*	4	0.8
*Echinopora*	3	0.6
*Mycedium*	3	0.6
*Euphyllia*	2	0.4
*Fungia*	2	0.4
*Herpolitha*	2	0.4
*Montastrea*	2	0.4
*Acropora*	1	0.2
	**531**	

## Discussion

### Water quality

The water temperature range at the natural reef site was greater than that reported for almost all reefs elsewhere except those of the Arabian Gulf [21–23]. The temperature range on the artificial reef was likely to have been similar to that on the natural reef despite its slightly greater depth (6.5 m MSL vs. 4.5 m MSL). Extended periods of high temperature during summer are significant stressors to the local coral communities, judging by the extensive coral bleaching at the natural reef site in February–March 2008, when temperatures were slightly higher than normal.

The turbidity regime was characterised by moderate background turbidity levels punctuated by intermittent events that increased turbidity to high levels (for coral reefs), often for several days. We are uncertain of the extent to which this turbidity regime, and the associated sedimentation, may have affected the health and resilience of corals at our study site. Certainly, a layer of sediment was present on abiotic surfaces during each visit to the site.

Erftemeijer et al. [24] emphasise the difficulty in defining what a ‘problem’ level of turbidity or sedimentation might be for any specific coral community, and the need to relate the definition of stress to local conditions, sediment characteristics and specific coral taxa. As a point of reference, Cooper et al. [25] suggest that long-term turbidity in excess of three NTU may cause sublethal stress to corals in coastal environments of the Great Barrier Reef. Although the 2.4 NTU average daily median in our data sits below that value, the frequent high turbidity events may be significant stressors, particularly when they coincide with high temperatures over summer.

### Settlement tiles

Coral settlement was recorded in all five deployments, including the single summer deployment in 2011/2012. Settlement rates to tiles deployed on the artificial reef and the natural reef were generally within the range 100-400 per m^-2^ over a spawning period, which falls within the typical range of 100-1000 recruits m^-2^ recorded in other studies using *terra* *cotta* tiles on Indo-Pacific reefs [26–31], although below the average number found on Great Barrier Reef sites [32]. Therefore we consider larval supply is unlikely to be a major constraint to coral community development on the artificial reef—or at least not to a greater extent than on many Indo-Pacific reefs.

Although the coarse taxonomy of the tile data limits comparisons with the quadrat data, the abundance of the ‘other’ category on the tiles appears to carry through to the quadrats as a predominance of *Pseudosiderastrea*, Faviidae, *Turbinaria* and *Mycedium* amongst juvenile corals. Very few of the settling Acroporidae or Pocilloporidae appear to survive to a size which might represent a reproductive coral, either on the artificial reef or the natural reef. This contrasts with some nearshore Queensland reefs with apparently similar environmental settings to our study site, where adult *Acropora* colonies are abundant [33]. In Dampier, Acroporidae and Pocilloporidae appear better suited to clear water environments further from the mainland, where they often comprise a significant component of the adult community [13].

### Quadrats

Coral density in the quadrats was far lower than on the tiles, as expected given the limitations of in-situ counts versus microscope counts, the likely high mortality of newly settled corals [34–37] and the presence on the artificial reef of sponges and soft corals capable of allelopathic exclusion [38,39]. On first inspection there was an apparent correlation between the tile and quadrat data—the 2009 trough and 2011 peak in mean coral density on the tiles perhaps corresponding to the 2010 trough and 2012 peak in mean coral density within the quadrats (Figures 4 and 6). However, the correlation was not evident at the subsite scale, where settlement density on the tiles bore no consistent relationship to subsequent recruit density in the quadrats. We conclude that either a) patterns of recruit density on the artificial reef are determined more by post-settlement mortality than settlement density, or b) settlement to the tiles is an unreliable indicator of settlement to the artificial reef.

Statistically significant coral distribution patterns in the quadrat data include differences in coral density between substrates (concrete > rock), aspects (seaward > landward), locations (east and central > west, landward side only) and surface orientations (horizontal > vertical). As the mechanisms behind these relationships were not investigated it is not possible to link cause and effect from our data. Some speculative influences on the observed distributions are outlined below.

The greater density of coral recruits on concrete than rock was a relatively late development, and is not a particularly strong trend, given the variability observed between surveys (Figure 8). If the trend is real, it indicates differences in the nature and/or rate of post-immersion processes between the two substrates. Concrete used in the reef had been exposed to aerial weathering for more than ten years, suggesting that its chemistry would have stabilised prior to immersion and would not have changed significantly after immersion. Differing rates of biofilm development, which are known to influence coral settlement [40,41], are perhaps the most likely explanation for different trajectories of coral density on the two substrates.

The effects of aspect and location on coral density —i.e. greater density on the seaward side of the reef and on the central and eastern locations—appear to be due to differential post-settlement survival, because the tile data displayed a greater density to landward than seaward, and no significant effect of location. The greater coral density on horizontal surfaces than vertical surfaces was a consistent pattern throughout the study. We interpret this relationship to result primarily from the pre-emption of space on vertical substrates by encrusting sponges and colonial invertebrates. Other explanations such as restricted light availability on vertical surfaces are feasible but we believe less likely, because coral colonies on the vertical surfaces are, on average, larger than those on the horizontal surfaces, indicating that the vertical surfaces are not inherently poor coral habitat. It seems the few colonies that manage to settle and survive on the vertical surfaces are able to thrive there, perhaps due to the relatively low sedimentation rate. The higher sedimentation rate on the horizontal surfaces is probably detrimental to corals, but may be even more detrimental to filter feeding invertebrates. In this scenario corals on the horizontal surfaces may actually benefit from sedimentation because they appear to withstand it better than their invertebrate competitors [42].

The current coral community composition on the artificial reef differs markedly from that of the nearby natural reef. The most obvious differences are the abundance of *Pseudosiderastreatayami* *,* *Mycediumelephantotus* and *Leptastrea* *purpurea* , and the scarcity of *Porites*, non-*Leptastrea* faviids and mussids on the artificial reef relative to the natural reef. We interpret these differences primarily as demographic traits; *P* *. tayami* , *M* *. elephantotus* and *L* *. purpurea* appear to be early colonisers whereas *Porites*, non-*Leptastrea* faviids and mussids recruit in lower numbers but may survive and grow to become abundant over the longer term.

It remains to be seen whether coral community composition and diversity on the artificial reef will eventually approach that of the natural reef. In most reported cases artificial reef communities do not closely resemble the adjacent natural communities, even after several decades [43,44]. However, the differences can usually be attributed to morphological and environmental differences between the artificial and natural reefs [43–45]. Perkol-Finkel et al. [46] suggest that, if artificial reefs and natural reefs are structurally similar, the artificial reef community is likely to eventually resemble the natural community. If this is the case in Dampier, the coral community of the Parker Point artificial reef should eventually resemble that of the nearby natural reefs, because the topographic relief of the artificial reef is similar to that of the natural reefs, and the igneous boulders that constitute the bulk of the artificial reef are similar to the natural rock substrate. The natural reef site we surveyed is two metres shallower than the artificial reef, which could potentially cause some divergence in coral community composition due to differences in depth-dependent factors including water temperature, light availability, wave energy and sedimentation. However, we have not observed such vertical stratification of coral community composition on any nearshore Dampier reefs. Therefore we believe that the coral community composition and diversity of the artificial reef should eventually approach that of the natural reef site, and that their current differences reflect their different stages of development.

Coral cover on the artificial reef increased rapidly in the last two years of the study (Figure 10). This is probably a geometric effect, reflecting the fact that growing coral colonies add progressively greater increments of surface area per unit time [47]. Survivorship is an important aspect of the rapid increase in cover, because older, larger colonies make the greatest contribution to total cover [47]. Colony survivorship on the artificial reef appeared to increase with size, such that most of the larger (> 20 mm diameter) colonies survived through each survey, enlarging over time. *Leptastrea* *purpurea* was by far the greatest contributor to coral cover because it was both abundant and fast-growing. In contrast, the slow growth rate and small size of *P* *. tayami* preclude it from contributing significantly to coral cover on the artificial reef, despite its numerical dominance.

The current rate of increase of coral cover on the artificial reef must eventually slow. Physical mechanisms that may slow or reverse the trend include disturbances such as cyclones, high or low water temperature excursions and high turbidity/sedimentation events (although corals on the artificial reef have so far survived several such events without apparent effects). Biological mechanisms include disease, predation and competition. Observations on the natural reef suggest that multiple mechanisms operate to constrain coral cover. Cyclone damage was evidenced by displacement or removal of entire colonies, but this affected only a minor proportion of colonies. Partial colony mortality was much more common, occurring across all coral taxa. In some cases it was clearly due to competitive interaction where coral colonies grew into close contact, but it was also common among colonies that were not in close contact. The cause(s) of mortality in these colonies was rarely determined, but a few instances of predation by *Drupellacornus* and bleaching-related mortality were recorded [16]. We presume the mechanisms constraining coral cover on the natural reef will likewise eventually constrain coral cover on the artificial reef.

The steady increase in coral density and cover on the Parker Point artificial reef to date is an encouraging sign that natural regenerative processes in Dampier Harbour remain adequate to restore lost coral habitat, where substrate and water quality permit, and to generate new coral habitat if that option is appropriate. We do not advocate artificial reef construction as a general solution to anthropogenic coral loss; restoring damaged reefs to their natural state is far preferable and should be attempted wherever possible [48]. However, the Parker Point example indicates that, in situations where the original substrate is lost and there is no restoration option, artificial reef construction is a viable alternative means of creating coral habitat.
